# Early seizures in acute stroke

**DOI:** 10.11604/pamj.2015.20.136.5925

**Published:** 2015-02-17

**Authors:** Chraa Mohamed, Najib Kissani

**Affiliations:** 1 Neurology Department, Mohamed VI University Hospital, Marrakesh, Morocco

**Keywords:** Ischemic stroke, early seizures, risk factors, prognosis

## Abstract

Early seizures (ES) may complicate the clinical course of patients with acute stroke. The aim of this study was to assess the frequency and the predictive factors for early seizures as well the clinical outcome in patients with first-ever stroke. A total of 352 consecutive patients with first-ever stroke, admitted to our department, were included in this retrospective study. Early seizures were defined as seizures occurring within 7 days from acute stroke. Patients with history of epilepsy were excluded. About 47 patients (13%) had early seizure, and 8 had a status epilepticus. We had 28 women and 19 men. The mean age was 71.6 ± 14.6. They were significantly more common in patients with cortical involvement, severe and large stroke, and in patient with cortical associated hemorrhage. ES were associated with an increase in adverse outcome (mortality and disability). Early seizures occured in about 13% of patients with acute stroke. In these patients hemorrhagic transformation is a predictive factor for ES. ES seem to be associated with a worse outcome after acute stroke.

## Introduction

Seizures are common during the early phase after stroke and have been reported to occur with a frequency of 2.5% to 5.7% within 7 to 14 days (the window where seizures are classified as early ones) of the acute episode [1]. In another hand stroke is the most common cause of epileptic seizures in the elderly population [2]. The effect of ES on prognosis is not known. If ES worsens prognosis, the prophylactic use of anticonvulsants in patients at risk is recommended [3]. Cortical involvement, stroke severity, multiple CT-scan lesions, hemorrhagic stroke, and are the most reported predictive factors [4, 5]. The aim of this study was to determine to rate, the predictor factors as well as the outcomes of ES in ischemic stroke.

## Methods

A total of 352 consecutive patients with first-ever stroke, admitted to the neurology department, Mohammed VI universitary hospital in Marrakesh, from January 2004 to December 2009, were included in this retrospective study. Early seizures were defined as seizures occurring within 7 days from acute stroke according to the international league against epilepsy guidelines (1993). Patients with history of epilepsy, transient ischemic attack, subarachnoid hemorrhage were excluded, as well as those with cerebral vein thrombosis. The assessment of stroke was performed by a senior neurologist. All patients performed a CT scan or MRI in some cases. Information extracted from the folders included age, sex, occupation, time for initial admission, clinical features, presence or absence of risk factors such as hypertension, diabetes mellitus, history of cigarette smoking and a history of stroke or epilepsy in the patient. Stroke severity was evaluated at admission using the National Institute of Health Stroke Scale (NIHSS). All analyses were performed with the SPSS (Property of Caddi Ayad university) and Statistical significance was set at p < 0.05.

## Results

A total of 352 patients with first ever ischemic stroke were included in this study. ES developed in 47 (13%) cases. The mean age was 60.5 ± 10.5 years with only 3 patients under the age of 45. Twenty-eight of the patients with ES (60%) were female and nineteen (40%) were male. The ES were simple in 39 cases (83%), and 8 patients had a status epilepticus. Risk factors of ischemic stroke are summarised in Table 1, it shows no significant differences between the groups of patients with and without ES. In a univariate analysis, large ischemic stroke, cortical involvement as well as hemorrhagic transformation were all more frequent in patients with ES than those without it (58.7% vs 37.4%, 68% vs 36% and 15% vs 7% respectively), see Table 1. The univariate analysis also showed that the initial stroke severity on admission was significantly higher in patients with ES than in patients without ES (p = 0.04). No significant differences were observed in terms of the major aetiologies of stroke (cardioembolic stroke and atherosclerosis) between the two groups. Finally the outcome was influenced by the ES in terms of mortality (47% in ES patients’ vs 26% in the other group) but not in terms of physical handicap (19% vs 18%).

**Table 1:** Clinical features in patients with and without early seizures

Variables	ES		Univariate analysis p
	Yes	No	
	47	305	
Mean age	60.5 ± 10.5	62.3 ± 8.5	
Hypertension	23 (49%)	153 (50%)	
Diabetes	9 (19%)	67 (22%)	
Ischemic heart desease	9 (19%)	52 (17%)	
Atrial fibrillation	7 (15%)	77 (25%)	
Hyperlipidemia	11 (23%)	69 (23%)	
Smoking	19 (40%)	104 (34%)	
Previous TIA	2 (4%)	9 (3%)	
Hemorrhagic transformation	7 (15%)	22 (7%)	0.03
large size	58.7%	37.4%	0.04
Cortical involvement	32 (68%)	105 (36%)	0.009
NIHSS (score)	13.6 ± 6.2	18.2±5.5	0.05

## Discussion

The pathophysiology of seizures after stroke is not completely understood but several mechanisms are hypothesized: cellular biochemical dysfunction with membrane instability of injured cells; transient excitoxic neurotransmitter release, such as glutamate, secondary to hypoxia; free radical damage, transient depolarizations of the ischemic penumbra with a resulting electrical irritable tissue [6, 7]. In our study, there was no relationship between ES and age, gender, ischemic heart disease, or hypertension, but a relationship was found between ES and initial stroke severity, these results are consistent with the conclusions of previous publications [4]. Some previous studies have indicated a relationship between infarct size and ES [8], results obtained in our study are in favor of the existence of such a relationship. In another hand we found a strong association between cortical involvement and ES in stroke, this observation has been reported in some studies [9–12], but not in others [13], this may be explained by acute cortical irritability, which may not undergo the same physiopathology as vascular epilepsy. We identified hemorrhagic transformation as associated with higher risk of ES in patient with stroke; these results are consistent with previous studies suggestions [13]. No significant differences were established between patients with and without ES in term of the underlying etiologies especially cardioembolic stroke which is reported to be a predictor for ES development [14, 15], which join the results of others similar studies [10, 11, 13]. The influence of ES on prognosis of stroke is controversial, in our study we found that ES were associated with higher risk of mortality but not of disability, our results join others conclusions in some studies [12], but not in others [13].

## Conclusion

About 13% of patient with stroke developed ES in our study. The predictive factors for ES occurrence in stroke were its clinical severity, cortical involvement and hemorrhagic transformation. Finally, ES was associated with high risk of mortality.
